# Associations between Distal Upper Extremity Job Physical Factors and Psychosocial Measures in a Pooled Study

**DOI:** 10.1155/2015/643192

**Published:** 2015-10-18

**Authors:** Matthew S. Thiese, Kurt T. Hegmann, Jay Kapellusch, Andrew Merryweather, Stephen Bao, Barbara Silverstein, Arun Garg

**Affiliations:** ^1^Rocky Mountain Center for Occupational and Environmental Health (RMCOEH), University of Utah, Salt Lake City, UT 84108, USA; ^2^Center for Ergonomics, University of Wisconsin-Milwaukee, Milwaukee, WI 53201, USA; ^3^Department of Mechanical Engineering, University of Utah, Salt Lake City, UT 84112, USA; ^4^Safety and Health Assessment and Research for Prevention (SHARP) Program, Washington State Department of Labor and Industries, Olympia, WA 98504, USA

## Abstract

*Introduction*. There is an increasing body of literature relating musculoskeletal diseases to both job physical exposures and psychosocial outcomes. Relationships between job physical exposure measures and psychosocial factors have not been well examined or quantified. These exploratory analyses evaluate relationships between quantified exposures and psychosocial outcomes. *Methods*. Individualized quantification of duration, repetition, and force and composite scores of the Strain Index (SI) and the Threshold Limit Value for Hand Activity Level (TLV for HAL) were compared to 10 psychosocial measures. Relationships and predicted probabilities were assessed using ordered logistic regression. Analyses were adjusted for age, BMI, and gender. *Results and Discussion*. Among 1834 study participants there were multiple statistically significant relationships. In general, as duration, repetition, and force increased, psychosocial factors worsened. However, general health and mental exhaustion improved with increasing job exposures. Depression was most strongly associated with increased repetition, while physical exhaustion was most strongly associated with increased force. SI and TLV for HAL were significantly related to multiple psychosocial factors. These relationships persisted after adjustment for strong confounders. *Conclusion*. This study quantified multiple associations between job physical exposures and occupational and nonoccupational psychosocial factors. Further research is needed to quantify the impacts on occupational health outcomes.

## 1. Introduction

Upper extremity musculoskeletal disorders (UEMSDs) are amongst the most prevalent and costly compensated disorders in worker's compensation systems. In Washington sate, work-related musculoskeletal disorders of the upper extremity and spine occurring without discrete trauma have been estimated to encompass 42.5% of compensable claims and lost time claims, respectively [1]. The highest rates of reported hand/wrist musculoskeletal disorders have been in the construction and manufacturing industries [1, 2].

A study from Washington state using the North American Industry Classification System (NAICS) with the Prevention Index reported that, among the top 25 industries, the highest median compensable costs per worker's compensation claim from 2002 to 2010 were in construction ranging from $11,280 to $30,101 [3]. Manufacturing costs per claim ranged from $8,869 to $10,914. Median costs in the services sector ranged from $5,687 to $10,053 [3].

Despite high prevalence rates, underreporting of injuries is reportedly widespread in the US and France [4]. A recent systematic analysis found that 90% of employers underreport occupational injuries and illnesses in Washington state [5]. The strongest predictors of underreporting included operating multiple shifts and use of the data for supervisor or respondent's job performance.

There have been reports investigating relationships between psychosocial factors and work-related musculoskeletal disorders, many of which include low back and neck pain [6–15]. These reports have detailed relationships between musculoskeletal outcomes and both work-organizational and personal psychosocial factors. Bongers et al. reported that a range of job organizational factors were related to both back pain and neck pain across the literature [9]. Another study found that multiple work organizational factors were associated with neck, shoulder, and low back pain, with strongest associations found with neck pain [12]. Only a few studies have investigated relationships between psychosocial factors and UEMSDS.

While there is increasing recognition of the importance of psychosocial factors in occupational health and safety, reports of psychosocial factors in relation to job physical factors for UEMSDs are uncommon. The most commonly reported associations are between surgical carpal tunnel syndrome (CTS) patient's disability and/or pain outcomes with depression [16, 17], pain anxiety [18, 19], and catastrophization [20, 21]. Depression and pain anxiety, but not neuroticism, are associated with worse upper extremity function in UEMSD patients that include some CTS patients [22]. However, whether the outcomes of surgical or medical case series mirror those in populations of workers is largely unknown.

Studies of association between psychosocial factors and increased risk of UEMSDs are limited and provide conflicting evidence. A prospective cohort study of CTS reported high job strain and low social support reported CTS risks [23], while a second report of the same cohort found mostly negative results from analyses of workplace psychosocial factors such as decision latitude [24]. One cross-sectional study of workers found associations between CTS and both job dissatisfaction and high job demands [25]. Another study of workers with UEMSDs that included a minority of CTS cases reported risks including low decision authority, high psychological demand, and low supervisor support, although job satisfaction and affective disorders were not reported [26]. There was no relationship between hand symptoms and job dissatisfaction in a study of hand therapists [27]. Job dissatisfaction and poorer physical health have been associated with CTS in a case-control study [6]; however, a prospective cohort study found no association between job satisfaction and new UEMSDs [28]. A study among French workers found job dissatisfaction to be weakly associated with symptoms only CTS case definition in a cross-sectional study without measured job exposure factors. That study also found that low job control was associated with one of two statistical models and psychological and psychosomatic “problems” are associated with CTS [29].

In contrast with psychosocial factors, there is an increasing body of literature prospectively quantifying relationships between job physical measurements and carpal tunnel syndrome [23, 24, 30–36], trigger digit [32], and lateral epicondylalgia [37] using measurement tools such as the American Conference of Governmental Industrial Hygienists (ACGIH), Threshold Limit Value for Hand Activity Level (TLV for HAL), and Strain Index (SI). In those studies, psychosocial factors have been largely treated as potential confounders, without assessment of magnitude of relationships and/or potential interactions between job physical exposures and psychosocial factors.

The objectives of this report are to perform exploratory analyses for potential relationships between job physical measures including (a) the TLV for HAL, (b) the SI, and (c) measures of force and repetition, with the psychosocial factors of job satisfaction, coworker support, supervisor support, physical exhaustion, mental exhaustion, anxiety, depressive symptoms, and general health. The general hypothesis is that increasing job physical exposures (e.g., higher force, higher repetition) will be associated with worsening of psychosocial responses.

## 2. Materials and Methods

This pooled study was approved by the Institutional Review Boards of Washington State, University of Wisconsin-Milwaukee and the University of Utah. Detailed descriptions of methods and data collection instruments used in this study are available and have been previously published [31, 32, 37, 38]; thus, abbreviated methods follow.

This study includes workers recruited from 35 diverse facilities representing 25 industries located in Illinois, Utah, Washington, and Wisconsin. These employees performed jobs in the manufacturing, food processing, healthcare, and office sectors. All workers provided written, informed consented prior to enrollment.

### 2.1. Psychosocial Factors and Demographic Data

Psychosocial factors and demographic data, including medical history, were collected using electronic questionnaires. Body mass indices were calculated from measured heights and weights. All data were collected by trained researchers who were blinded to the job physical exposures of the workers.

A total of 10 psychosocial measures were common between all three research sites. These included (1) general health compared to others, (2) depressive symptoms, (3) physical exhaustion after work, (4) mental exhaustion after work, (5) how well participants get along with coworkers, (6) job satisfaction, (7) how well participants get along with their closest or immediate supervisor, (8) degree to which participants would recommend their job to others, (9) if participants would take the job again, and (10) degree to which participants feel that their employer cares about their health and safety on the job. Responses were categorized into 3 or 4 levels (Table 1). Questions 1, 2, and 10 were adapted from the NIOSH Generic Job Stress Questionnaire [39], and questions 6, 8, and 9 were adapted from the Job Content Questionnaire [40]. The other questions were developed by the research team for this study. While these questions have been used in other studies [41–43], they have not been validated. We were unable to include extensive batteries of questions due to enrollment time limits and having participants excessively removed from production jobs.

### 2.2. Job Physical Exposures

Individual data used to calculate the TLV for HAL [44, 45] and SI [31, 44, 46] were collected by trained ergonomics analysts who were blinded to symptoms and health data. Job physical raw data included (a) videotapes of tasks, (b) analyst peak hand force rating [47], (c) individual task duration, and (d) length of work shift.

Videos were analyzed to extract data of analyst's overall force ratings, exertion durations, postures, and work speed. Exertion, duration, and repetition were also assessed directly from recorded video of multiple cycles of each participant's tasks. Expert ergonomists who were specifically trained and standardized viewed each video and quantified individual duration of exertions, repetition, and overall force ratings for both hands of each worker for SI score calculations. Trained ergonomics analysts took video recordings and provided hand-specific peak force ratings (using the Borg CR-10 scale) for each task performed by each worker. Video recordings were later analyzed in laboratory to quantify (i) Borg CR-10 force ratings for each sub-task, (ii) verbal anchor HAL ratings [48], (iii) total frequency of exertion, (iv) frequency of forceful exertions, (v) total percent duration of exertion, (vi) percent duration of forceful exertions, and (vii) posture and speed of work used to calculate SI scores. Forceful exertions were defined as those rated as “light” or greater on the Borg CR-10 scale (i.e., Borg CR-10 ≥ 2). Analysts were blinded to the health and psychosocial status of the workers.

Exertion requirements measured included (i) verbal anchor scale for HAL rating [45, 48], (ii) counts of efforts per minute, and (iii) % duration of exertion [46]. Methods to determine efforts per minute, % duration of exertion, work speed, and posture were published previously [46].

### 2.3. SI and TLV for HAL Scoring and Components

TLV for HAL and SI were calculated for each task that a worker performed. TLV for HAL scores were calculated using the ACGIH method as follows: Score = [Analyst Peak Force Rating on Borg CR-10 Scale/(10 − HAL Rating)]. We treated TLV for HAL score as a continuous variable. TLV for HAL was also categorized using the ACGIH prescribed cut-points: below the Action Limit (AL) (score < 0.56), between the AL and Threshold Limit Value (TLV) (0.56 ≤ score ≤ 0.78), and above the TLV (score > 0.78). Calculation of SI scores followed prior published methods and incorporated the analyst's overall force rating, counts of efforts/min, % duration of exertion, posture, work speed, and task duration [46]. First, SI was treated as a continuous variable. Then, SI score was categorized into low risk (SI ≤ 6.1) and high risk (SI > 6.1) based on the most recent recommendation by Moore et al. [49]. TLV for HAL and SI were calculated for each task that a worker performed. TLV for HAL scores were calculated as follows: Score = [Analyst Peak Force Rating on Borg CR-10 Scale/(10 − HAL Rating)]. SI scores were calculated in the manner described by Moore and Garg [46] using total efforts per minute and total percent duration of exertion.

A large proportion of workers (*n* = 710, 38.7%) performed multiple tasks as part of their job. We defined “typical exposure” (i.e., exposure from the task the worker performed for the largest percentage of a work shift) as being representative of the worker's daily exposure. For comparative purposes we also explored the alternative techniques of “peak exposure” (i.e., exposure from the most stressful task performed) and time-weighted-average (TWA) exposure from all tasks performed during a work shift. Details of these job physical exposure summarization techniques are described elsewhere [31, 44].

### 2.4. Statistical Analyses

Ordered logistic regression was performed to assess the risk between worker physical exposures and psychosocial factors. All analyses were performed using SAS 9.4 software (Cary, NC). Statistical significance was at *P* < 0.05. All models included age, gender, and body mass index (BMI) as potential confounders.

For interpretive purposes, we also calculated predicted probabilities of participants being in a given psychosocial category per unit of meaningful change in a given job physical exposure measure. Meaningful changes in physical exposure were defined as 4 efforts per minute, 5% duration of exertion, 1 Borg CR-10 unit of force, 0.1 units of TLV for HAL score, and 3 units of Strain Index score.

Analyses were treated as exploratory and thus no corrections were made to the models or results to account for multiple comparisons.

## 3. Results and Discussion

A total of 1834 participants were included in this pooled analysis. Most (59.8%) were female (see Table 1) with a mean age of 41.1 years and mean BMI of 28.7 kg/m^2^. Most (60.2%) had never smoked tobacco and relatively few had been diagnosed with diabetes mellitus (4.7%) or thyroid problems (6.2%). Job physical exposure measures for the typical job task were similar for both the left and right hands and thus only right hand data and results are reported (Table 1). Frequency and percentage of the 10 psychosocial questions assessed show reasonable distribution across this pooled sample of workers (Table 2).

Several associations between quantified job physical exposures and psychosocial factors were identified (Table 3). Similarly, there were strong associations between age, gender, and BMI and all psychosocial measures except willingness to take the job again and recommending the job to others. Both the TLV for HAL and the Strain Index were associated with job satisfaction, supervisor support, whether a worker would recommend the job to someone else and how likely the worker would be to take the job again (*P* ≤ 0.05). The TLV for HAL additionally was associated with physical exhaustion after work and whether the employer was thought to care about the worker's health and safety on the job (*P* ≤ 0.01). The only psychosocial factor associated with the Strain Index but not the TLV for HAL was mental exhaustion after work (*P* ≤ 0.01). Neither model showed association with general health status, feelings of depression, or supervisor support (*P* > 0.17). Other measures of physical exposure similarly showed broad association with multiple psychosocial factors. In general, peak force and forceful duration were more strongly associated with more psychosocial outcomes than other exposure measures. Forceful duration of exertion was associated with (*P* ≤ 0.05) or tending towards association with (*P* ≤ 0.20) all psychosocial outcomes.

The directionality of most of the relationships between physical exposure and psychosocial outcomes was as hypothesized, where an increase in job physical exposure measure (e.g., higher force, higher repetition, and higher duration of exertion) was associated with a worsening of psychosocial response (e.g., more physical exhaustion, less job satisfaction, and less likely to take this job again). Exceptions were for general health and mental exhaustion where increasing job physical exposures tended to be associated with better psychosocial responses (e.g., better general health, less mental exhaustion).

For comparative purposes, analyses were performed evaluating relationships between both peak and TWA job physical exposure summarization techniques and the results were essentially identical to typical job physical exposure measures (data not shown).

**Figure 1:**
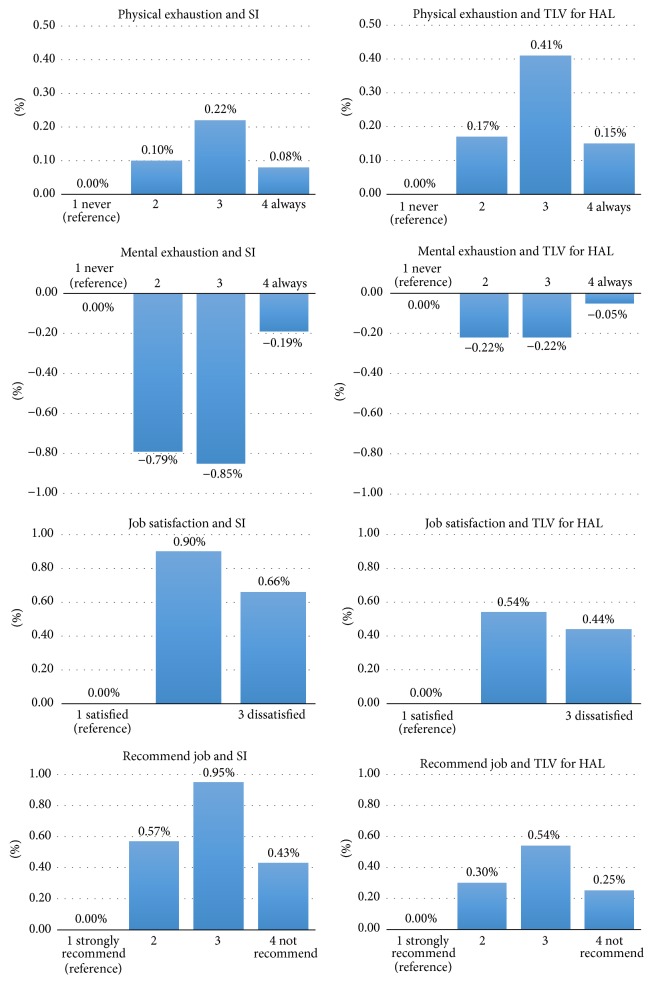
Estimates in change of likelihood for a worker with mean age and BMI to be in a worse psychosocial category with a unit icrease in job physical exposure measure for the typical job as compared to the change in the best psychosocial category (unit change for SI = 3, unit change for TLV for HAL = 0.1).

Figure 1 represents estimates in change of likelihood for a worker of mean age and BMI to be in a worse psychosocial category per unit increase in job physical exposure measure for the typical job as compared to the probability in the best psychosocial category. This figure demonstrates both directionality and magnitude of the relationships between SI or TLV for HAL and psychosocial factors. For example, consider TLV for HAL rated exposure and reporting being physically exhausted; for each 0.1 units increase in TLV for HAL there is a 0.17% increased probability that an average worker will report seldom being exhausted, 0.41% increased probability they will report often being physically exhausted, and 0.15% increased probability they are reporting always being exhausted as compared to those reporting never being physically exhausted. When comparing differences between SI and TLV for HAL, the directionality and relative relationships are similar for most psychosocial outcomes. Differences in magnitude (*y*-axis) between SI and TLV for HAL measures might simply be the result of unit differences for estimating probabilities.

## 4. Discussion

The results of this study show a relatively consistent statistical association between increased job physical exposure and worsening of psychosocial outcomes notwithstanding the noteworthy exceptions of general health compared to others and mental exhaustion after work which showed generally more positive responses associated with higher physical exposures. Many prior studies have evaluated relationships between psychosocial factors and WMSDs; however, this is the first study that we are aware of to assess relationships between job physical factors and psychosocial outcomes.

While most associations are consistent, such as poorer responses to job satisfaction, recommending job to others, and taking job again, as exposures increase, there are a few associations that stand out as potentially unique. For example, perhaps unsurprisingly, perceived physical exhaustion appears to be most strongly related to force, but not necessarily repetition. Conversely and somewhat unexpectedly, depression appears to be most strongly related to repetition, but not force. This might suggest that more monotonous work somehow provokes depressive symptoms. Perhaps contradictorily, to the seemingly consistent association between job dissatisfaction and increased physical exposures, the tendency of workers to report relatively* better* general health and* less* mental exhaustion with increased job physical exposures suggests that at least moderately strenuous jobs may somehow be beneficial to one's perceived well-being (if not job satisfaction).

It is important to note that while the statistical associations between job physical exposures and certain psychosocial factors appear very strong, the relative impact on probability of response is relatively modest (Figure 1). This implies that there are likely several factors, other than physical exposures, that influence the psychosocial state of manufacturing workers. Thus, psychosocial factors should continue to be studied as possible independent risk factors for occupational injuries and illnesses, such as CTS.

Only a few studies evaluating relationships between psychosocial factors and UEMSDs have been able to statistically control the potential confounder of job physical factors [11, 15] or have created theoretical constructs that account for job both physical factors and psychosocial factors in the etiological pathway for UEMSDs [12, 13]. The psychosocial factors assessed in the literature have focused on both work-organizational (e.g., job pace, job control, and job satisfaction) factors and personal (e.g., depressive symptoms or anxiety) factors. The paper by Huang et al. theorized about the potential causal pathways and relationships between job physical factors, psychosocial factors, and health outcomes [14]. Several studies have found statistical relationships between different measures of psychosocial factors, while statistically controlling for job physical exposures; however, there has not been an established relationship between job physical factors and psychosocial factors. To the best of our knowledge, this is the first study to quantify the relationship between these two domains.

Study strengths include a large, multicenter study including workers from 4 diverse states that used highly comparable study methods. Workers also were enrolled from a wide diversity of occupations and spectrum of job physical factors. The broad range of job physical factors suggests the study is reasonably powered to detect relationships based on those factors. Data collection instruments used identical or nearly identical measures. Questionnaires, psychosocial measures, health status, and job measurements were obtained in all workers, regardless of symptoms. The job measurement teams and health measurement teams were blinded to each other.

Study limitations include the exploratory and cross-sectional nature of this study which limit the study to hypothesis generation regarding potential associations. The workers were mostly in manufacturing, which may limit extrapolations to other industrial sectors. The healthy worker effect may have had some impact, although the enrollments intentionally sought workers regardless of symptoms. The non-Gaussian distribution of the answers to the psychosocial factors likely somewhat limits the power to detect effects, especially for feelings of depression and coworker support. The number of psychosocial factors is also somewhat limited, although generally more robust than prior reports. Additionally, not all psychosocial measures were validated.

## 5. Conclusion

These analyses demonstrate multiple relationships between job physical exposure measures and psychosocial outcomes after adjustment for age, BMI, and gender. Higher job physical exposures appear to elicit consistently worse responses to job satisfaction, willingness to take the job again, and recommending the jobs to others. Depressive symptoms appear to be more strongly related to increasing repetition measures alone, while perceived physical exhaustion appears to be more strongly related to force measures alone. Conversely, higher physical exposure results in relatively* better *perceived general health and mental exhaustion, implying that at least moderately demanding work may have a positive psychological effect. Ultimately, these findings should help future researchers as they attempt to quantify associations between psychosocial factors and various occupational injuries and illnesses.

## Figures and Tables

**Table 1:** Descriptive statistics for the pooled population and job physical factors for the typical job on the right hand (*n* = 1834).

	Mean ± SD or *n* (%)	Min–max
Age (years)	41.13 ± 11.34	18.0–72.0
Female gender	1096 (59.8%)	
Body mass index (kg/m^2^)	28.67 ± 6.49	15.9–58.6
Never smoke tobacco	1104 (60.2%)	
Diabetes mellitus	87 (4.7%)	
Thyroid problems	114 (6.2%)	
Total duration (%)	66.1 ± 22.4	0–100
Forceful duration (%)	40.5 ± 31.3	0–100
Total exertions (per minute)	22.2 ± 16.7	0–112.9
Forceful exertions (per minute)	13.8 ± 15.7	0–111.3
Hand activity level rating	3.4 ± 1.9	0–7.9
Overall force (Borg rating)	2.3 ± 1.6	0–9
Peak force (Borg rating)	2.7 ± 1.7	0–10
Strain Index	6.7 ± 9.9	0.3–117.0
TLV for HAL	0.64 ± 0.63	0.00–8.00

**Table 2:** Descriptive statistics for the psychosocial factors for the pooled population.

How is your general health compared to people your own age	
1 better	263 (14.3%)
2	631 (34.1%)
3	743 (40.5%)
4 worse	197 (10.7%)
How often do you feel down, blue, or depressed	
1 never	505 (27.5%)
2	1028 (56.1%)
3	269 (14.7%)
4 always	32 (1.7%)
Physically exhausted after work	
1 never	212 (11.6%)
2	873 (47.6%)
3	562 (30.6%)
4 always	187 (10.2%)
Mentally exhausted after work	
1 never	474 (25.9%)
2	882 (48.1%)
3	397 (21.7%)
4 always	81 (4.4%)
Get along with your coworkers	
1 always/often	947 (51.6%)
2	745 (40.6%)
3 hardly ever/never	142 (7.7%)
Job satisfaction	
1 satisfied	521 (28.4%)
2	941 (51.3%)
3 dissatisfied	372 (20.3%)
How often does your supervisor demonstrate appreciation for the work you do	
1 always	1207 (65.8%)
2	527 (28.7%)
3 never	100 (5.5%)
How likely would you recommend your job to someone else	
1 strongly recommend	278 (15.2%)
2	894 (48.8%)
3	435 (23.7%)
4 not recommend	227 (12.4%)
How likely would you take this job again	
1 very likely	521 (28.4%)
2	721 (39.3%)
3	429 (23.4%)
4 unlikely	163 (8.9%)
My employer cares about my health and safety on the job	
1 strongly agree	450 (24.5%)
2	1140 (62.2%)
3	176 (9.6%)
4 strongly disagree	68 (3.7%)

**Table 3:** Statistical significance of ordinal logistic regression results analyzing relationships between typical job physical exposure measures in the right hand and psychosocial factors after adjustment for age, gender, and body mass index.

Exposure	General health	Down, blue, or depressed	Physically exhausted	Mentally exhausted	Job satisfaction	Supervisor support	Get along with your coworkers	Recommend job	Take this job again	Employer cares
Age	<0.001^∗∗^	<0.001^∗∗^	<0.001^∗∗^	0.007^∗∗^	0.004^∗∗^	0.033^∗^	<0.001^∗∗^	0.227	0.595	0.027^∗^
BMI	<0.001^∗∗^	<0.001^∗∗^	<0.001^∗∗^	0.008^∗∗^	0.004^∗∗^	0.033^∗^	<0.001^∗∗^	0.223	0.594	0.029^∗^
Gender	<0.001^∗∗^	<0.001^∗∗^	<0.001^∗∗^	0.007^∗∗^	<0.004^∗∗^	0.034^∗^	<0.001^∗∗^	0.232	0.005^∗∗^	0.026^∗^
Total duration	0.006^∗∗^	0.121	0.459	<0.031^∗∗^	<0.001^∗∗^	0.028^∗^	0.067^∗^	<0.001^∗∗^	<0.005^∗∗^	0.178
Forceful duration	0.006^∗∗^	0.091	0.178	<0.001^∗∗^	<0.001^∗∗^	0.028^∗^	0.017^∗^	<0.001^∗∗^	<0.001^∗∗^	0.089
Total repetition	0.147	0.001^∗∗^	0.714	0.172	<0.001^∗∗^	0.456	0.468	0.096	0.001^∗∗^	0.295
Forceful repetition	0.176	0.001^∗∗^	0.198	0.008^∗∗^	<0.001^∗∗^	0.383	0.518	0.003^∗∗^	<0.001^∗∗^	0.096
HAL	0.065	0.001^∗∗^	0.739	0.020^∗^	<0.001^∗∗^	0.031^∗^	0.101	0.010^∗^	<0.001^∗∗^	0.099
Overall force	0.589	0.829	0.003^∗∗^	0.019^∗^	0.006^∗∗^	0.787	<0.001^∗∗^	<0.001^∗∗^	0.002^∗∗^	0.002^∗∗^
Peak force	0.075	0.868	<0.001^∗∗^	0.013^∗^	<0.001^∗∗^	0.073	0.005^∗∗^	<0.001^∗∗^	<0.001^∗∗^	<0.001^∗∗^
Strain index	0.681	0.166	0.402	0.002^∗∗^	0.002^∗∗^	0.228	0.617	0.001^∗∗^	0.012^∗^	0.769
TLV for HAL	0.439	0.213	0.006^∗∗^	0.118	<0.001^∗∗^	0.292	0.031^∗^	<0.001^∗∗^	0.041^∗^	<0.001^∗∗^

^∗^0.05 ≥ *P* > 0.01; ^∗∗^0.01 ≥ *P*.
